# Antimalarial efficacy test of the aqueous crude leaf extract of *Coriandrum sativum* Linn.: an *in vivo* multiple model experimental study in mice infected with *Plasmodium berghei*

**DOI:** 10.1186/s12906-024-04577-0

**Published:** 2024-07-12

**Authors:** Getu Habte, Sisay Habte, Oda Jilo, Wondwosen Alemu, Kedir Eyasu, Welela Meka, Getabalew Shifera, Wubishet Gezimu, Milkias Dugasa, Sanbato Tamiru, Meta Mamo, Abiyot Kelecha

**Affiliations:** 1 Department of Pharmacology and Clinical Pharmacy, School of Pharmacy, College of Health Sciences, Addis Ababa University, P.O. Box 1176, Addis Ababa, Ethiopia; 2 Department of Pharmacy, College of Health Sciences, Mattu University, P.O. Box 318, Mettu, Ethiopia; 3 Department of Biology, College of Natural and Computational Sciences, Ambo University, P.O. Box 19, Ambo, Ethiopia; 4 Department of Computer Science, College of Engineering and Technology, Mattu University, P.O. Box 318, Mettu, Ethiopia; 5 Department of Chemistry, College of Natural and Computational Sciences, Mattu University, P.O.Box 318, Mettu, Ethiopia; 6 Department of Nursing, College of Health Sciences, Mattu University, P.O. Box 318, Mettu, Ethiopia

**Keywords:** *Coriandrum sativum*, Antimalarial, Chemosuppressive, *In vivo*, *Plasmodium berghei*, Curative, Prophylaxis, Extract, Multiple-models, Efficacy

## Abstract

**Background:**

Malaria continues to wreak havoc on the well-being of the community. Resistant parasites are jeopardizing the treatment. This is a wake-up call for better medications. Folk plants are the key starting point for antimalarial drug discovery. After crushing and mixing the leaves of *Coriandrum sativum* with water, one cup of tea is drunk daily for a duration of three to five days as a remedy for malaria by local folks in Ethiopia. Additionally, *in vitro* experiments conducted on the plant leaf extract elsewhere have also demonstrated the plant’s malaria parasite inhibitory effect. There has been no pharmacologic research to assert this endowment in animals, though. This experiment was aimed at evaluating the antimalarial efficacy of *C. sativum* in *Plasmodium* *berghei* infected mice.

**Methods:**

The plant's leaf was extracted using maceration with distilled water. The extract was examined for potential acute toxicity. An evaluation of secondary phytoconstituents was done. Standard antimalarial screening models (prophylactic, chemosuppressive, curative tests) were utilized to assess the antiplasmodial effect. In each test, thirty mice were organized into groups of five. To the three categories, the test substance was given at doses of 100, 200 and 400 mg/kg/day before or after the commencement of *P. berghei* infection. Positive and negative control mice were provided Chloroquine and distilled water, respectively. Rectal temperature, parasitemia, body weight, survival time and packed cell volume were ultimately assessed. Analysis of the data was performed using Statistical Package for Social Sciences.

**Results:**

No toxicity was manifested in mice. The extract demonstrated a significant inhibition of parasitemia (*p* < 0.05) in all the models. The inhibition of parasite load was highest with the upper dose in the suppressive test (82.74%) followed by the curative procedure (78.49%). Likewise, inhibition of hypothermia, weight loss hampering, improved survival and protection against hemolysis were elicited by the extract.

**Conclusions:**

The results of our experimental study revealed that the aqueous crude leaf extract of *C. sativum* exhibits significant antimalarial efficacy in multiple *in vivo* models involving mice infected with *P. berghei*. Given this promising therapeutic attribute, in depth investigation on the plant is recommended.

## Background

Malaria is a devastating blood-born zoonotic protozoal disease that damages the red blood cells of the victims [1, 2]. It is found at the top of dangerous fatal infections caused by the protozoan parasites. The five main plasmodial protozoans responsible for causing malaria include *P. Knowlesi*, *P. malariae*, *P. vivax*, *P. falciparum* and *P. ovate *[1]. These protozoan parasites are mainly propagated from human to human through the bite of pregnant Anopheles mosquitoes. Additionally, infections from *P. knowlesi* could be contracted from monkeys infected with the parasite through transmission by the aforementioned vectors [3, 4]. In rare scenarios, the disease can also be transmitted through blood transfusion and the needles contaminated by the parasite. In all the infection types, the pathologic mechanism behind is the rupture of red blood cells, the release of inflammatory mediators, and toxic chemicals that result in the symptoms of malaria such as shaking chills. The ultimate consequence of this phenomenon is the death of the patient from hypoxia unless appropriate measures are commenced [3, 5, 6].

Despite relentless efforts to contain malaria, the disease continues to persistently endanger the life of inhabitants dwelling in the areas endemic to the disease as well as visitors to these areas [1, 7, 8]. Eighty-four countries of the world are still endemic to the disease, albeit a lot of triumphs have been gained through the planned engagement of the World Health Organization (WHO) and its compassionate partners [9, 10]. The main severely affected regions of the world are the developing countries whereas the developed regions of Europe, North America, Australia, and China contract the infection when they have a journey to the areas endemic to the disease [1, 10, 11]. Once in history, these regions were malaria endemic before they cut it out. The WHO reported recently that the cases and deaths from malaria is escalating from year to year: 2019; 227 million cases, 568, 000 deaths [12], 2020; 241 million cases, 625,000 deaths [13], 2021; 247 million cases, 619, 000 deaths [9], and 2022; 249 million cases, 608,000 deaths [1], each consecutive year, accounting for more than half a million deaths.

About 95% of malaria cases and 96% of deaths is seen within the population residing in sub-Saharan Africa. Every minute, a child under 5 years is put in to death by malaria in this region accounting for about 80% of malaria deaths [1, 9, 14]. In addition, pregnant women living in sub-Saharan Africa are vulnerable to the danger of malaria. 32% of pregnancies (out of 42 million) in this region were affected by malaria according to the WHO malaria report. Maternal anemia, low birth weight, maternal and infant mortality and congenital infection are the adverse consequences encountered from malaria during pregnancy [10, 15, 16]. Lack of infrastructure, scarcity of medicines and preventive tools, mosquitos’ resistance to preventive chemicals, inadequacies of the approved vaccine, and more importantly continued loss of parasite sensitivity to antimalarial drugs contribute to malaria’s damage in this region [1, 10, 17, 18].

Over time, plasmodial protozoan loss of sensitivity to the commonly employed drugs in the management of malaria is imposing a tremendous challenge to contain the disease [19, 20]. Chloroquine (CQ) was once the drug of choice for the management of malaria due to *P. falciparum* all over the world [21, 22]. Similarly, these parasites were responding to the contemporary Sulfadoxine-Pyrimethamine combination which was among the recommended treatment regimens. However, due to selection of the resistant plasmodial parasites to these armaments, at present, they lost their gain to mitigate the infection of *P. falciparum*. Artemisinin group of drugs are the most active antimalarial drugs commenced today against the severe form of plasmodial parasite, *P. falciparum *[19, 20, 23, 24]*.* These weapons are the contributions from Chinese folk medicine where Professor Tu Youyou scientifically unveiled the ammo, and rescued the lives of millions of malaria endemic communities. This innovation is recognized as the priceless gift from the Chinese community to the world [25, 26]. Nevertheless, reports from different parts of the world, including the recent report from Ethiopia [27], is alerting us that plasmodial parasites gained resistance traits to this life saving advancement [22, 24, 28, 29].

In response to this wake-up call, thorough scientific investigations on plants with historical background of medicinal value in a community is of paramount importance [30, 31]. Folk plants are the key starting point for antimalarial drug discovery [32] as evidenced by the previous development of medicines such as quinine and artemisinin from cinchona and Artemisia plants, respectively [26, 33, 34]. *C. sativum* is in a list of plants utilized by the Ethiopian community for traditionally managing malaria [35]. As a consequence, the plant is a candidate for scientific exploration to develop antimalarial medicines with better efficacy and safety profile [36].

**Fig. 1:**
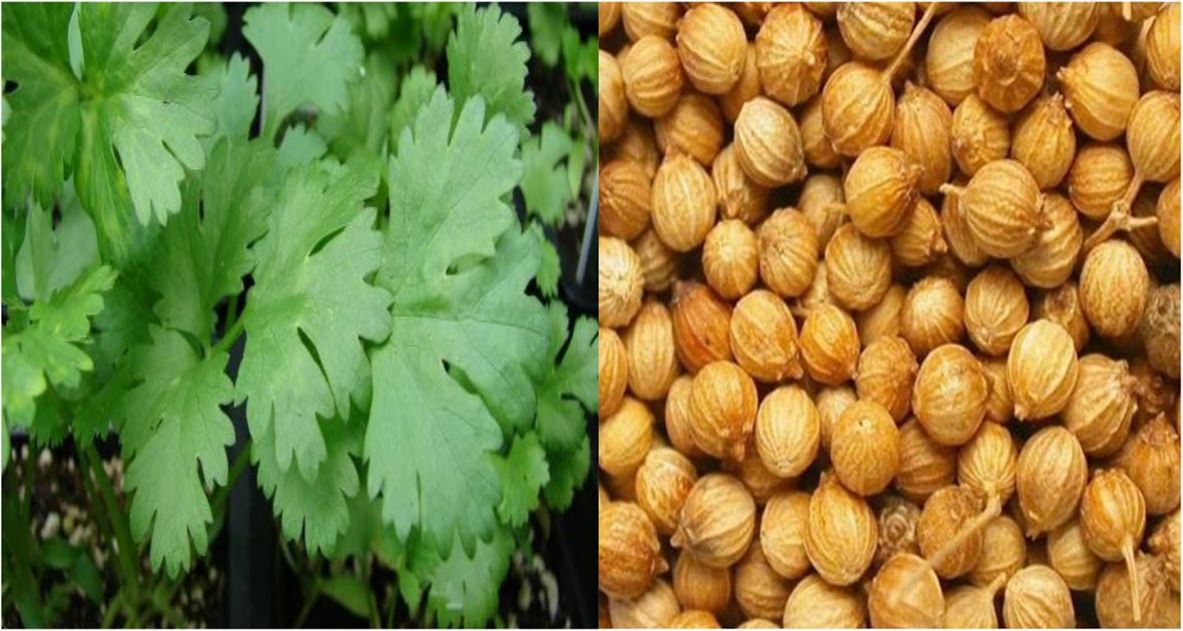
Picture of the coriander plant, *Coriandrum sativum Linn*

*C. sativum* (Fig. 1) [37] is a herb commonly utilized for seasoning purposes as a spice and is known by the name coriander in English [38]. Different communities assigned varying vernacular names to the plant according to their vicinity, culture and language. For instance, Coriandre in French, Koriander in German, Coriandolo in Italian, kuzbara in Arabic, Dhania in Hindi, Cilantro in Spanish, Pak chee in Thai and Wan-sui in Mandarin Chinese. In Ethiopia, it is known by the name Dimbelal [35]. Taxonomically, *C. sativum* is grouped under the family Umbelliferae (Apiaceae). The plant is said to have originated in the Mediterranean region. Asia, North America, Europe and other parts of the world then gained the plant through cultivation [39, 40].

Throughout the world, coriander has applications in food and preparations of medicinal value [41, 42]. The leaves, seeds and roots of this culinary plant are edible by virtue of its astonishing flavor and aroma [43]. Linoleic acid, dihydrocoriandrine and coriandrine are components that are responsible for the plant’s attractive scene as a condiment [37, 40]. The use of coriander as part of folk traditional remedies traced back to the early civilizations, around 1550 BC. Each plant part (leaf, seed, root) has been used for ameliorating different varieties of ailments. Previous studies on the plant affirmed its anti-diabetic effect, antioxidant activity, anxiety relief, and analgesic properties [37, 44, 45].

Furthermore, there are well documented plant’s antimicrobial activity including antiprotozoal [46, 47], antifungal, antibacterial [48, 49], antihelminths [50, 51], and larvicidal [52] effects. Current drugs such as Tetracyclines which possess antiprotozoal and antibacterial activity have also antimalarial effect, suggesting plant’s potential antimalarial efficacy [53]. *In vitro* experimentations conducted on the *C. sativum* plant leaf extract elsewhere also advocated the plant’s malaria parasite inhibitory effect [54, 55]. In addition, previous *in vitro* and *in vivo* investigations done on the other plants in the same family (Umbelliferae, or Apiaceae) showed antimalarial activity[56, 57]. Furthermore, the folk’s use of the plant as antimalarial remedy has also been published. More specifically, in Ethiopian folk medicine, after crushing and mixing the leaves of *C. sativum* with water, one cup of tea is drunk daily for the duration of three to five days as a remedy for malaria [35]. These all provided an excellent basement for further conduction of the in vivo antimalarial test to assert this arrogated endowment employing experimental animals. This insight being the trigger, the aim of the current experimental work was to evaluate the antimalarial efficacy of *C. sativum* in mice infected with *P. berghie*.

## Methods

### Plant sample collection

The *C. sativum* leaves were collected from Alle District of Ilubabor Zone, Southwest Ethiopia. The plant sample was appropriately wrapped using newspaper, and then supplied to the Ethiopian National Herbarium which is the body of the College of Natural and Computational Sciences of Addis Ababa University, Ethiopia, in November 2022. Here, the identification of the sample was carried out by the taxonomist (Mr. Melaku Wondafrash), and reference number GH04/2022 was assigned to the specimen. For the sake of an antimalarial pharmacologic experimental assay, the plant material was further collected from the aforementioned site where the community utilizes it as a traditional remedy in malaria-infected individuals.

### Extraction procedure

The gathered leaf of the plant was first thoroughly washed using tap water, and then cleaned utilizing gauze to remove any leftover dirt and dust. Then, it was carefully dried at room temperature away from direct sunlight. Afterward, the dried leaf was sliced into pieces with the help of a mortar and pestle producing coarsely powdered material (800mg). Next, the plant's coarsely powdered leaf was extracted using a cold maceration process with distilled water which was conducted at a room temperature (20 -25 C°), employing the method of Habte et al. slightly modifying it [58].

As per the method, in the beginning, 400mg of the coarsely powdered leaf of the plant was immersed in 1L of distilled water in an Erlenmeyer flask. After that, the flask was put on the mechanical shaker working at a speed of 120rpm to hasten the extraction phenomenon. The shaker was occasionally operated with on and off times over 72 h of extraction. Following the 3 days process of extraction, the Erlenmeyer flask was removed from the shaker and rested on the table. Then after, the distillate having the water extract of the plant was initially separated from the heavy marc using gauze. Under suction filtration, then, the extract was further subjected to partitioning with the help of Whatman filter paper. To the marc, twice, fresh distilled water was added and the same extraction procedure was repeated.

The other half coarse powder (400mg) was also extracted following the same technique mentioned above. The filtrates from the overall procedure were then combined in a round bottom flask, and concentrated employing a lyophilizer at -50C° (freezing), -10 C° (sublimation), and 10 C° (desorption). Last but not least, the extraction yield (%) was determined, and the concentrated plant extract was kept in a refrigerator at -20 °C. The formula illustrated herein below was employed to calculate the yield [59].

1 $$Percentage\;extraction\;yield=\frac{weight\;of\;concentrated\;extract}{total\;macerated\;weight\;of\;coarsely\;powdered\;leaf}\ast100$$

### The infecting parasite and employed animals

The mice species known as Swiss albino mice, having a weight range of 25 to 28g, aged between 6 and 8 weeks, were used in the present research work. These mice were obtained from the Ethiopian Public Health Institute (EPHI). The study was conducted in Mattu University, Pharmacy Department laboratory between November and December 2022. For the aim of acclimatization to the environment of actual experimentation, the mice stayed in the laboratory for a week without any investigation done on them. Polypropylene cages were utilized for harboring the laboratory animals. The mice were maintained in a standard room with good airflow and temperature with a twelve-hour light–dark cycle. The animals were fed with a standard animal pellet diet, and water was provided ad libitum.

*P. berghei* (ANKA strain sensitive to CQ) infected donor mice were obtained from EPHI. Every week, then, the parasites were conserved by successive transmission of blood from plasmodium-infected donor mice to immaculate mice having no pathogen of interest. This passage of the parasites continued until the parasitemia in newly infected donors got optimum, 30–37% [60]. To determine the level of parasitemia reached, 0.5 to 1 mm tip of the tails of the mice were cut using scissors. The microscope was used to find out the level of parasitemia. The blood from the mice reaching the parasitemia level of 30–37% was used for infecting the mice in the efficacy tests in the three models. The animals were euthanized by employing cervical dislocation after getting unconscious with a single intraperitoneal injection of sodium pentobarbital anesthesia (60 mg/kg body weight). The experimental theatre employed in this work was following the international guideline for studies utilizing animals, and in accordance with the National Academy of Sciences Guide for the care and use of laboratory animals [61]. In addition, from Pharmacy Department, Research and Technical Review Committee, Mattu University, a letter stating permission of the experimental work, with a reference number ‘Pharm 2019/17’, was obtained.

### Animal group designation and dosing

In each model (suppressive, curative, prophylactic), thirty male mice were employed. The mice utilized in each model were divided into five groups containing six mice in separate cages randomly. The first groups of mice were given distilled water (10ml/kg/day) with the help of oral gavage and acted as negative controls. The second, third, and fourth groups received the leaf extract at escalating doses of 100 mg/kg, 200 mg/kg, 400 mg/kg/day respectively by the same route as mentioned for the negative control mice. The last three groups of mice were administered CQ at 10 mg/kg/day by mouth with the aid of oral gavage (positive control group). The test substances and the controls were provided to the mice once daily for four consecutive days. The doses of the extract administrated to the experimental groups were decided based on the acute toxicity investigation findings, and preliminary tests done on the plant leaf extract.

### Plant’s safety establishment on acute toxicity

For the purpose of establishment of the plant extract’s safety, the well-accepted international guideline, the Organization for Economic Cooperation and Development (OECD) guideline 425, was utilized for determination of the plant’s oral acute toxicity [62]. In accordance with the guideline, at the beginning, a single female mouse was fasted overnight and weighed and provided a dose of extract at 2000mg/kg orally via gavage. Then, the mouse was kept away from food for extra 2 h. Following that, the mouse was under close monitoring for the next 24 h. Due to the absence of signs of toxicity and death, extra four female mice were provided with the same dosage of the plant extract. In a nutshell, the mice were monitored over two weeks for behavioral deviations and physical aberrations including lacrimation, appetite loss, erection of hair, convulsions and tremors, and incidence of deaths.

### Screening plant’s malaria ameliorating potential in mice

#### Early infection model

Evaluating the plant’s antimalarial efficacy in early infection was assessed by employing the four-day suppressive test previously illustrated by Peter et al. [63], and adopted by Habte et al. [64] with a slight adjustment. The procedure begins with sacrificing the donor mice, mentioned in section ‘the infecting parasite and employed animals’, reaching the parasitemia level of 30–37% for infecting the mice in the efficacy test. After the animals were sacrificed with cervical dislocation, cardiac puncture was carried out and the blood was collected into a falcon tube having anticoagulant 2% trisodium citrate. The samples of blood from all participating donor mice were then pooled together in an attempt to minimize the variations. The dilution was made with normal saline. The inoculum was prepared based on parasitemia of the donor mice blood, and red blood cell count of normal mice. Accordingly, a solution of blood containing 5 × 10^7^ infected RBCs per 1 ml of blood was prepared [65, 66].

The mice in each of the five groups for this model, explained in the section titled ‘animal group designation and dosing’ were first coded and marked. These mice were then intraperitoneally inoculated with 0.2 ml of blood having nearly 1 × 10^7^ RBCs infected with *P. berghei* on day one (D01). Then, after 3 h on the same day (D001) animals were fed with the test and control substances according to their group. Dosing continued on a daily bases for the other three consecutive days; day two (D002) after 24 h, day three (D003) after 48 h and day four (D004) after 72 h. Prior to the administration of substances to the mice, immediately before D001 (at D0) and/or 96 h after D001 (D4), at the end of day four, on day 5, parameters detailed in the upcoming subtitles were measured. The mice were also closely followed up for a month for determination of the days of survival [58, 60, 67].

#### Assessing the duration of survival of mice and level of parasitemia

To determine the level of parasitemia, blood sample was taken at D4 by cutting off the tip of the tail of each mouse using scissors. Non-greasy frosted slides were used to prepare thin blood films. The slides were stained using freshly prepared10% Giemsa for 15 min. The stain was then washed away with distilled water. After drying out, the slides were then examined with the aid of a compound microscope with the X100 objective. The number of PRBCs out of the total RBCs in the fields of the microscope were counted and utilized to determine the percentage parasitemia (PP). For each mouse in each group, two slides were stained and examined under the microscope. Again, for every slide seen under the microscope, three microscopic fields with estimated 200–500 cells were carefully counted. The following equation was then employed to calculate the PP for each mouse [64, 68, 69].

2 $$PP=\frac{The\;number\;of\;PRBCs}{The\;total\;RBCs\;in\;the\;fields\;of\;microscope}\times100$$

Using the hereinafter formula, the mean percentage parasitemia suppression (PPS) was calculated [58, 70]:

3 $$PPS=\frac{(mean\;PP\;of\;the\;negative\;control-mean\;PP\;of\;the\;experimental\;group)}{mean\;PP\;of\;the\;negative\;control}\times100$$

Each group’s mean survival time (MST) was decided applying the formula indicated hereunder [70]:

4 $$MST=\frac{The\;sum\;of\;time\;mice\;survived\;in\;days\;for\;each\;group}{total\;mice\;number\;in\;each\;group}$$

#### Mice weight, packed cell volume and temperature deviations from the baseline

The mouse rectal temperature and weight were taken immediately before the commencement of treatment at D0 and just after treatment at D4. After feeding into SPSS, data were checked for completeness and the software was run and the percentage change in mean was computed. In addition, packed cell volume (PCV) was determined for each mouse at a similar time interval mentioned herein for rectal temperature and weight. In an attempt to determine PCV, first, the tip of the mouse tail was cut off using scissors and blood was drawn into microhaematocrit capillary tubes (heparinized). After filling the capillary tubes with blood to 75% of their height, the end facing opposite to the blood collecting opening (dry end) was closed with the sealing clay. After that, the capillary tubes were taken to a micro-hematocrit centrifuge and put in it where the unsealed end faced inside. The machine operated at 11,000 rpm for duration of five minutes. Next, after quitting the rotating centrifuge, the capillary tubes were removed and PCV was determined by applying the following standard formula [65]:

5 $$PCV=\frac{volume\;of\;RBCs\;in\;centrifuged\;blood}{total\;volume\;of\;centrifuged\;blood}X100$$

#### Established infection model

The assessment of the aqueous crude leaf extract of *C. sativum* plant curative antiplasmodial efficacy in the established infection was carried out following Rane’s test as earlier described by Ryley and Peters, with a few adjustments [71, 72]. First of all, the inoculum was prepared in the same way as illustrated for the four-day suppressive test as indicated under the section ‘early infection model’. Next, the animals (five groups for this model), explained in the section titled ‘animal group designation and dosing’ were coded, marked and intraperitoneally inoculated with 0.2 ml of blood having nearly 1 × 10^7^ RBCs infected with *P. berghei* on day one (D01). 72 h after, on day 4 (D001), animals were fed with the test and control substances according to their group. Dosing continued on a daily bases for the other three consecutive days; day five (D002) after 96 h, day six (D003) after 120 h and day seven (D004) after 144 h. Prior to administration of substances to the mice, immediately before D001 (at D0) and/or 168 h after D01 (D4), at the end of day seven, on day 8, parameters detailed in the upcoming subtitles were measured. The mice were also closely followed up for a month for determination of the days of survival.

#### Mice survival time and parasitemia determination

At the end of the seventh day since the start of inoculation of the parasite, at D4, by cutting the apex of the tail of each mouse with scissors, blood was removed and thin blood films were prepared using non-greasy frosted slides. The slides were examined with the aid of microscope as explained under the section entitled ‘early infection model; assessing the duration of survival of mice and level of parasitemia’. Furthermore, PP and PPS were determined by employing Eqs. 2 and 3 mentioned in the text. Mice were followed up for 30 days, and eventually, the mice MST was determined by the formula illustrated in Eq. 4.

#### Changes in mice packed cell volume, temperature, and weight

On day five, prior to initiation of dosing of animals, PCV, temperature, and weight of each mouse were measured. Additionally, twenty-four hours after the final doses were administered, on the seventh day of inoculation, similar measures were taken. Moreover, PCV was measured according to the formula given in Eq. 4. Data gathered from the mice were then organized and processed in similar way as demonstrated under the section entitled ‘early infection model; mice weight, PCV and temperature deviations from the baseline’.

#### Chemopreventive model

Evaluation of the *C. sativum* leaf extract’s antimalarial prophylactic activity against the development of the infection was assessed by employing the repository test previously described by Peters with slight adjustments [66, 69, 73]. At the beginning, the randomly grouped experimental mice, the two control groups and three test groups, as mentioned earlier in this text under the section entitled ‘animal group designation and dosing’ were coded, marked and given the crude plant leaf extract and the negative and positive control substances as per their designation on day one (D001). The animals were daily provided the extract and the control substances for the extra subsequent three days: day two (D002) immediately after 24 h, day three (D003) immediately after 48 h and day four (D004) immediately after 72 h of D001.

Following this, the inoculum was prepared in a similar way as demonstrated in Peter’s four-day suppressive test procedure mentioned under the title ‘the early infection model’. Then, at the end of 96 h, immediately, on day five (D01), the mice were injected intraperitoneally 0.2 ml of blood having nearly 1 × 10^7^ RBCs infected with *P. berghei*. Further, prior to administration of substances to the mice, immediately before D001 (at D0) and/or 168 h after D001 (D4), at the end of day six, on day 7, parameters detailed in the upcoming subtitles were measured. The mice were also closely followed up for a month for determination of the days of survival.

#### Mice survival time and parasitemia determination

Counting 168 h from the beginning of dosing the animals with the extract and control substances, at the start of day 7 (D4), scissors were used to cut off the tip of the tail of mice to get blood for investigation to assess the blood level of plasmodial parasites. The same procedure detailed under the section entitled ‘early infection model; assessing the duration of survival of mice and level of parasitemia’ was duplicated for the demonstration of the procedure and determination of percent parasitemia and percent parasitemia suppression. Moreover, PP and PPS were determined by employing Eqs. 2 and 3 mentioned in the text. In addition, mice were monitored for 30 days, and finally, the mice MST was calculated before their sacrifice as described by the formula provided in Eq. 4.

#### Changes in mice packed cell volume, temperature, and weight

On day five, prior to initiation of the dosing of animals, PCV, temperature, and weight of each mouse were measured. Additionally, twenty-four hours after the final doses were administered, on the seventh day of inoculation, similar measures were taken. Moreover, PCV was measured according to the formula given in Eq. 4. Data gathered from the mice were then organized and processed in a similar way as demonstrated the in the section entitled ‘early infection model; mice weight, PCV and temperature deviations from the baseline’.

#### Evaluation of secondary phytoconstituents

Standard procedures were followed to establish the secondary phytochemical components of the aqueous leaf extract of *C. sativum* plant grown in Ethiopia [69, 74, 75]. Moreover, the phytochemicals were examined in qualitative and quantitative tests. Alkaloids, tannins, flavonoids, glycosides, steroids, saponins and terpenoids were among the secondary phytochemicals evaluated in the present study.

#### Analysis of the experimental data

The data collected in three of the screening models were organized. After fed in to the statistical package for social sciences (SPSS) software version 22, then it was analyzed. One way analysis of variance (ANOVA) followed by the Tukey post hoc test analytic step was applied to compare the mean differences. Accordingly, MST, PPS, changes in mean PCV, body weight, and rectal temperature of the mice infected with *P. berghei* between the extract-treated groups and the controls were compared. In addition, comparison was done among the extract-received groups in each model. Statistical significance level was set at a *P*-value not equal to or greater than 0.05 with confidence interval of 95%.

## Results

### Extraction yield

The extraction yield obtained from the aqueous crude leaf extract of *C. sativum* after macerating 800g of the powdered plant sample was 18.75%. Moreover, the extract’s appearance following the dry freezing concentration procedure was found to be brown-colored powder.

### Plant’s safety establishment on acute toxicity

Observation for gross behavioral changes and physical appearances in acute toxicity testing revealed that no obvious symptoms of acute poisoning were elicited by the plant extract. In addition, during the first-day single animal test, and throughout the acute toxicity study follow-up period (two weeks), the death of any of the experimental mice was not seen at the administered dose level of 2000 mg/kg.

### Plant’s antiplasmodial efficacy in the four-day suppressive model

#### Impact on mice blood parasite level

**Fig. 2:**
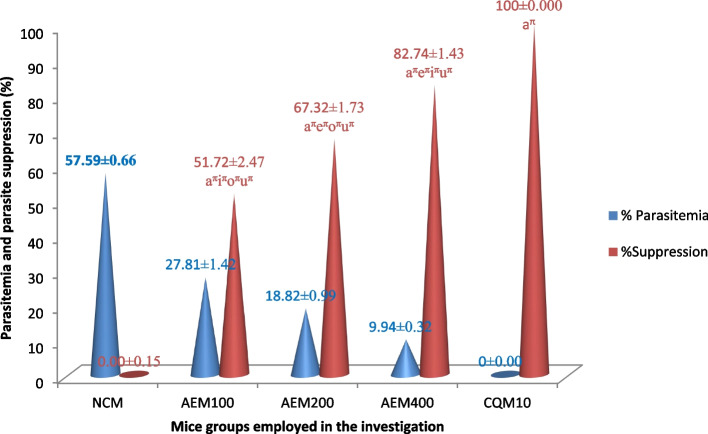
Measure of parasite suppressing potential of the aqueous crude leaf extract of *C. sativum* after infecting mice with *P. berghei* in the four day suppressive test. Data presentation is in the form of mean ± standard error of the mean (Number of mice included in each of the group = 6). NCM, mice group which received negative control, distilled water, 10ml/kg/day; AEM100, mice group which received aqueous crude leaf extract, 100mg/kg/day; AEM200, mice group which received aqueous crude leaf extract, 200mg/kg/day; AEM400, mice group which received aqueous crude leaf extract, 400mg/kg/day; CQM10, mice group which received positive control, chloroquine base, 10mg/kg/day; a, comparing with NCM; e, comparing with AEM100; i, comparing with AEM200; o, comparing with AEM400; u, comparing with CQM10; ^π^*p* < 0.05

Figure 2 summarizes the *C. sativum* plant aqueous crude leaf extract’s chemosuppressive activity on the level of blood plasmodium parasites in early infection. All the doses provided to the mice prevented the rise in blood parasite relative to the vehicle. The measure of percentage parasite suppression indicated that higher doses resulted in better plasmodial inhibition activity. The ceiling plasmodial suppression effect (82.74%) was elicited by the 400mg/kg/day of the extract. Despite its highest suppressive effect, this upper dose of the plant leaf extract was inferior to the standard antimalarial drug, CQ, which exterminated the parasite to zero blood level. Furthermore, the differences in halting the blood parasite level among all the doses of the extract, extracts and control groups, and among the control groups were statistically significant (*p* < 0.05).

#### Influence on the duration of survival of mice

**Fig. 3:**
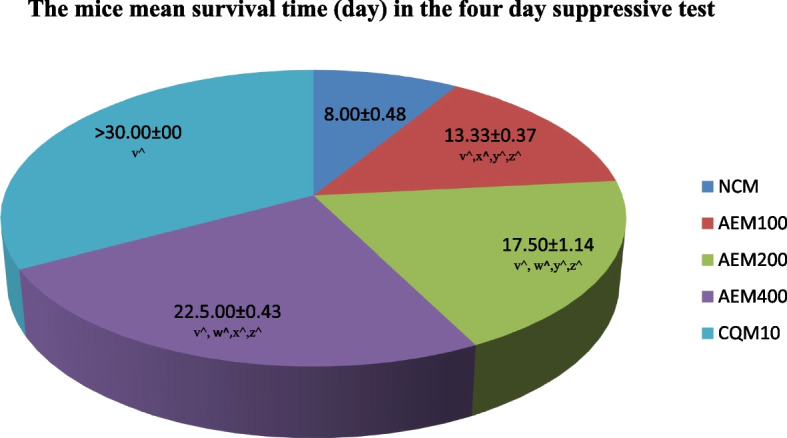
The measure of survival time (days) of mice after infection with *P. berghei*, and access to different doses of aqueous crude leaf extract of *C. sativum* in the four day suppressive test. Data presentation is in the form of mean ± standard error of the mean (Number of mice included in each of the group = 6). NCM, mice group which received negative control, distilled water, 10ml/kg/day; AEM100, mice group which received aqueous crude leaf extract, 100mg/kg/day; AEM200, mice group which received aqueous crude leaf extract, 200 mg/kg/day; AEM400, mice group which received aqueous crude leaf extract, 400 mg/kg/day; CQM10, mice group which received positive control, chloroquine base, 10mg/kg/day; v, comparing with NCM; w, comparing with AEM100; x, comparing with AEM200; y, comparing with AEM400; z, comparing with CQM10; ^*p* < 0.05. The mice were under close monitoring for a total of 30 days

The mice were closely followed for 30 days, and the average days of survival were determined per group as described in Fig. 3. The longest average survival time from the plant extract was 22.5 days, which was observed in the mice group that received the plant extract at the ceiling dose (400mg/kg/day). The ascending doses of the extract exerted longer survival time. Moreover, the mean survival days for mice among the groups is statistically significant (*p* < 0.05). Mice that received CQ survived 30 days and beyond.

#### Influence on temperature and weight measures

The aqueous crude leaf extract of the *C. sativum* plant inhibited the loss of weight occurring due to parasitemia development, and averted the decline in temperature of mice in the four-day antimalarial screening model (Table 1). Furthermore, the mean percentage weight decline was 5.02% with the extract at a dose of 400mg/kg/day whereas it was 12.95% with the placebo. As far as temperature is concerned, the percentage reduction was seen to be 6.65% and 1.16% for the negative control group and those that received the highest dose of the extract respectively. In all cases, however, the weight loss ameliorating effect and resistance to fall in body temperature were found to be the maximum with the standard antimalarial medication employed in this investigation.

**Table 1:** Body weight and rectal temperature changes of mice infected with *P. berghei*, and administered the aqueous crude leaf extract of *C. sativum* in the four day suppressive test

Group	Body Weight (g)			Rectal Temperature (°C)		
	D_0_	D_4_	Change (%)	D_0_	D_4_	Change (%)
NC	25.79 ± 0.77	22.45 ± 0.67	-12.95 ± 0.65	35.49 ± 0.17	33.13 ± 0.17	-6.65 ± 0.11
AE100	26.01 ± 0.37	23.31 ± 0.39	-10.40 ± 0.28a^π^i^π^o^π^u^π^	35.58 ± 0.25	34.29 ± 0.27	-3.62 ± 0.61a^π^i^π^o^π^z^π^
AE200	25.97 ± 0.74	24.53 ± 0.65	-5.51 ± 0.68a^π^e^π^o^π^u^π^	35.43 ± 0.20	34.66 ± 0.18	-2.18 ± 0.33a^π^e^π^o^π^z^π^
AE400	26.06 ± 0.69	25.42 ± 0.71	-2.40 ± 0.64a^π^e^π^i^π^u^π^	35.26 ± 0.30	35.10 ± 0.29	-0.45 ± 0.11a^π^e^π^i^π^o^π^
CQ10	26.00 ± 0.93	26.77 ± 0.97	2.95 ± 0.18a^π^	35.07 ± 0.26	36.38 ± 0.23	3.76 ± 0.22a^π^

Measurements are expressed as mean ± standard error of the mean. (the number of mice assigned in a group = 6). *D*_*0*_ pre-treatment measured value on day 0, *D*_*4*_ post-treatment measured value on day 4, *NC* Negative control, distilled water, 10 ml/kg/day, *AE* Aqueous crude leaf extract, *CQ* Chloroquine. a, comparing with NC; e, comparing with 100 mg/kg; i, comparing with 200 mg/kg; o, comparing with 400 mg/kg; u, comparing with CQ10; ^π^*p* < 0.05. The numbers after the acronyms in the column titled group (400, 200, 100, 10) indicate the doses given to those groups in mg/kg/day

#### Influence on packed cell volume measure

**Fig. 4:**
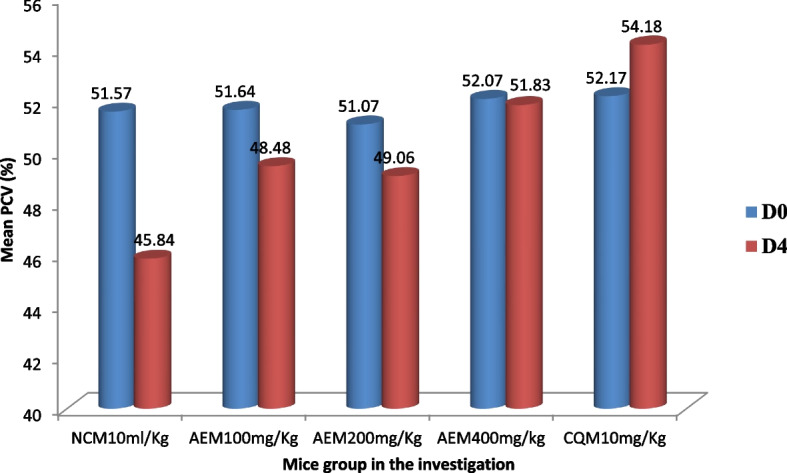
The measure of change of packed cell volume of *P. berghei* infected mice, and access to stepping up doses of aqueous crude leaf extract of *C. sativum* in the four day suppressive test. Data presentation is in the form of mean ± standard error of the mean (Number of mice included in each of the group = 6). D0, measure on day 0, pre-treatment; D4, measure on day 4, post-treatment. NCM10ml/kg, mice group which received negative control, distilled water, 10ml/kg/day; AEM100mg/kg, mice group which received aqueous crude leaf extract, 100mg/kg/day; AEM200mg/kg, mice group which received aqueous crude leaf extract, 200mg/kg/day; AEM400mg/kg, mice group which received aqueous crude leaf extract, 400mg/kg/day; CQM10mg/kg, mice group which received positive control, chloroquine base, 10mg/kg/day. PCV, packed cell volume. a, comparing with NCM10ml/kg; e, comparing with AEM100mg/kg; i, comparing with AEM200mg/kg; o, comparing with AEM400mg/kg; u, comparing with CQM10mg/kg; ^π^*p* < 0.05. Indicated in the rectangles is the mean percentage change of PCV between post-treatment and pre-treatment measures

The plant extract protected the malaria parasite-induced hemolysis of red blood cells in early infection as evidenced from the data described in Fig. 4. Each ascending dose of the *C. sativum* plant aqueous crude leaf extract inhibited the decline of PCV with the preceding dose being less beneficial. The lowest mean percent decline (0.046%) in PCV from the extract-received group was measured in mice that received the 400mg/kg/day. No mice in the CQ received group showed reduction in PCV measure from the base line. The percentage decline in PCV was compared for all the mice groups relative to the negative control mice and among themselves. In this regard, there was a statistically significant (*p* < 0.05) difference in halting the hemolysis compared to the control and among all the groups.

### Plant’s antiplasmodial efficacy in the curative model

#### Impact on mice blood parasite level

**Fig. 5:**
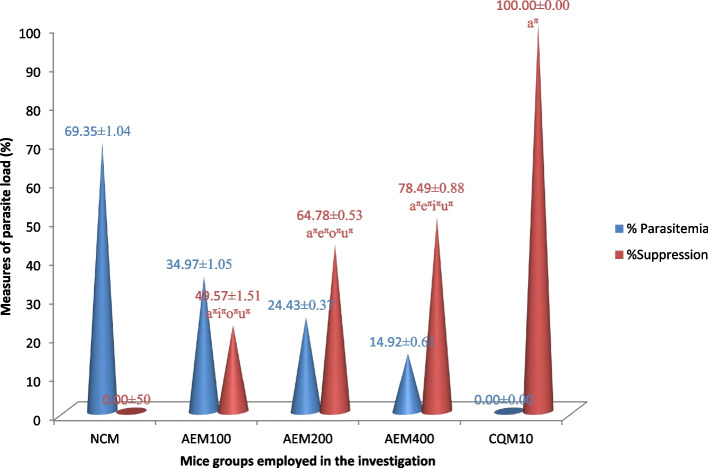
Measure of parasite load reverting potential of the aqueous crude leaf extract of *C. sativum* after infecting mice with *P. berghei* in the curative test. Data presentation is in the form of mean ± standard error of the mean (Number of mice included in each of the group = 6). NCM, mice group which received negative control, distilled water, 10ml/kg/day; AEM100, mice group which received aqueous crude leaf extract, 100mg/kg/day; AEM200, mice group which received aqueous crude leaf extract, 200mg/kg/day; AEM400, mice group which received aqueous crude leaf extract, 400mg/kg/day; CQM10, mice group which received positive control, chloroquine base, 10mg/kg/day; a, comparing with NCM; e, comparing with AEM100; i, comparing with AEM200; o, comparing with AEM400; u, comparing with CQM10; ^π^*p* < 0.05

The *C. sativum* plant aqueous crude leaf extract’s chemosuppressive effect on the established plasmodial infection is summarized in Fig. 5. The lower level of protozoal parasite load was seen with the doses of the investigational plant as compared to distilled water. The extract’s highest plasmodial suppressive capability was 78.49% which was observed at the upper dose level (400mg/kg/day). The parasite inhibition was in increasing order from the lowest dose of the plant extract towards the standard drug which completely eradicated the parasite and cured the mice. This escalating effect was also statistically significant (*p* < 0.05) among all the groups under the study.

#### Influence on the duration of survival of mice

**Fig. 6:**
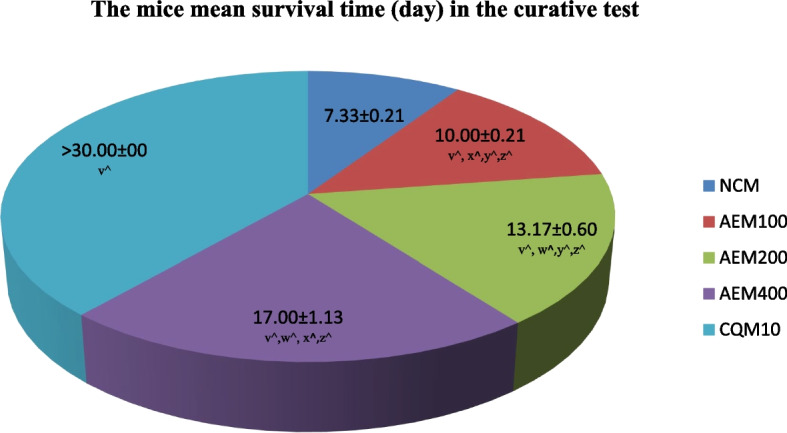
The measure of survival time (days) of mice after infection with *P. berghei*, and access to different doses of aqueous crude leaf extract of *C. sativum* in the curative test. Data presentation is in the form of mean ± standard error of the mean (Number of mice included in each of the group = 6). NCM, mice group which received negative control, distilled water, 10ml/kg/day; AEM100, mice group which received aqueous crude leaf extract, 100mg/kg/day; AEM200, mice group which received aqueous crude leaf extract, 200mg/kg/day; AEM400, mice group which received aqueous crude leaf extract, 400mg/kg/day; CQM10, mice group which received positive control, chloroquine base, 10mg/kg/day; v, comparing with NCM; w, comparing with AEM100; x, comparing with AEM200; y, comparing with AEM400; z, comparing with CQM10; ^*p* < 0.05. The mice were under close monitoring for a total of 30 days

Figure 6 summarizes the average days of survival of mice that were first infected with *P. berghei*, and then provided varying doses of aqueous crude leaf extract of *C. sativum* in the curative test. The test mice which took the plant extract at the larger doses survived more, and 17 days was the longest MST recorded. In spite of the fact that the mice receiving 400mg/kg/day dosage survived the longest days, the effect was inferior to that of mice in the group which received CQ.

#### Influence on temperature and weight measures

The malaria parasite triggered hypothermia relieving potential and weight loss ameliorating capacity of the water extract of *C. sativum* in the curative antimalarial screening model is illustrated herein in Table 2. CQ, the positive control medication employed in this investigation, showed no reduction in the mean weight and temperature of mice. Likewise, the extract at different ascending doses averted both weight loss and hypothermia. Nevertheless, even at the highest dose administered in this experiment, the plant didn’t completely prevent the mean reduction in both of the parameters mentioned here. The highest mean percentage temperature and weight change for the extract were 6.79% and 10.10% at 400 mg/kg/day respectively. Otherwise, the mean percentage change was downward in the mice which received the negative control, weight 10.37% and temperature 6.69%.

**Table 2:** Body weight and rectal temperature changes of mice infected with *P. berghei*, and given the aqueous crude leaf extract of *C. sativum* in the curative test

Group	Body Weight (g)			Rectal Temperature (°C)		
	D_0_	D_4_	Change (%)	D_0_	D_4_	Change (%)
NC	22.33 ± 0.31	20.02 ± 0.64	-10.37 ± 2.51	33.34 ± 0.15	30.03 ± 0.57	-9.94 ± 1.63
AE100	22.30 ± 0.49	21.75 ± 0.38	-2.38 ± 0.62a^π^i^π^o^π^u^π^	33.25 ± 0.16	31.97 ± 0.15	-3.85 ± 0.44a^π^i^π^o^π^u^π^
AE200	22.36 ± 0.26	23.82 ± 0.28	6.55 ± 1.15a^π^e^π^o^π^u^π^	33.24 ± 0.17	33.88 ± 0.21	1.96 ± 0.76a^π^e^π^o^π^u^π^
AE400	22.37 ± 0.34	25.78 ± 0.43	15.25 ± 1.13a^π^e^π^i^π^u^π^	33.34 ± 0.17	35.65 ± 0.21	6.96 ± 0.78a^π^e^π^i^π^u^π^
CQ10	22.42 ± 0.36	27.70 ± 0.37	23.72 ± 2.80a^π^	33.20 ± 0.07	37.15 ± 0.49	11.93 ± 1.49a^π^

Measurements are expressed as mean ± standard error of the mean. (the number of mice assigned in a group = 6). *D*_*0*_ pre-treatment measured value on day 0, *D*_*4*_ post-treatment measured value on day 4, *NC* Negative control, distilled water, 10 ml/kg/day, *AE* Aqueous crude leaf extract, *CQ* Chloroquine. a, comparing with NC; e, comparing with 100 mg/kg; i, comparing with 200 mg/kg; o, comparing with 400 mg/kg; u, comparing with CQ10; ^π^*p* < 0.05. The numbers after the acronyms in the column titled group (400, 200, 100, 10) indicate the doses given to those groups in mg/kg/day

#### Influence on packed cell volume measure

**Fig. 7:**
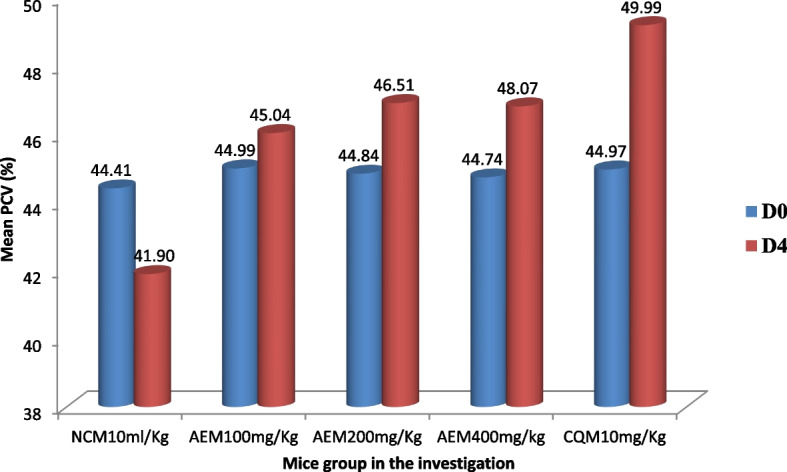
The measure of change of packed cell volume of *P. berghei* infected mice, and access to stepping up doses of aqueous crude leaf extract of *C. sativum* in the curative test. Data presentation is in the form of mean ± standard error of the mean (Number of mice included in each of the group = 6). D0, measure on day 0, pre-treatment; D4, measure on day 4, post-treatment. NCM10ml/kg, mice group which received negative control, distilled water, 10ml/kg/day; AEM100mg/kg, mice group which received aqueous crude leaf extract, 100mg/kg/day; AEM200mg/kg, mice group which received aqueous crude leaf extract, 200mg/kg/day; AEM400mg/kg, mice group which received aqueous crude leaf extract, 400 mg/kg/day; CQM10mg/kg, mice group which received positive control, chloroquine base, 10mg/kg/day. PCV, packed cell volume. a, comparing with NCM10ml/kg; e, comparing with AEM100mg/kg; i, comparing with AEM200mg/kg; o, comparing with AEM400mg/kg; u, comparing with CQM10mg/kg; ^π^*p* < 0.05. Indicated in the rectangles is the mean percentage change of PCV between post-treatment and pre-treatment measures

In Rane’s test for an established infection, the decline in the PCV from the baseline was observed only in the mice group which received the vehicle for reconstituting the extract (Fig. 7). The highest mean percent change in PCV was found to be 4.67% for the extract-received mice at the administered dose of 400mg/kg/day. The maximum parasite induced hemolysis reversing capability was seen with CQ (9.39%). A comparison of the various doses of the *C. sativum* plant aqueous crude leaf extract with the vehicle depicted that there was a statistically significant difference (*p* < 0.05) in preventing the loss of red blood cells.

### Plant’s antiplasmodial efficacy in the prophylactic model

#### Impact on mice blood parasite level

**Fig. 8:**
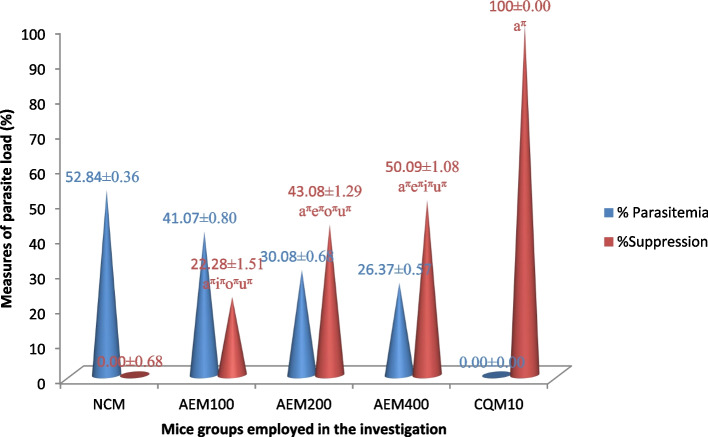
The parasite load of mice given the aqueous crude leaf extract of *C. sativum*, and then infected with *P. berghei* in the prophylactic test. Data presentation is in the form of mean ± standard error of the mean (Number of mice included in each of the group = 6). NCM, mice group which received negative control, distilled water, 10 ml/kg/day; AEM100, mice group which received aqueous crude leaf extract, 100mg/kg/day; AEM200, mice group which received aqueous crude leaf extract, 200mg/kg/day; AEM400, mice group which received aqueous crude leaf extract, 400mg/kg/day; CQM10, mice group which received positive control, chloroquine base, 10mg/kg/day; a, comparing with NCM; e, comparing with AEM100; i, comparing with AEM200; o, comparing with AEM400; u, comparing with CQM10; ^π^*p* < 0.05

The chemopreventive effect of the aqueous crude leaf extract of the *C. sativum* plant towards blood plasmodial parasites is presented in Fig. 8. In this prophylactic evaluation of the plant extract, the highest parasite suppressive effect recorded was 50.09% at 400mg/kg/day dosing. The other two doses of the crude extract also produced lower load of the parasites in the blood in comparison to the placebo. Moving from the vehicle through elevating doses of the extract towards CQ, the effect exerted was heightened and the difference in parasitemia suppression was statistically significant at *p* < 0.05. CQ, the standard medication employed as a positive control and comparator, absolutely prevented the development of parasitemia.

#### Influence on the duration of survival of mice

**Fig. 9:**
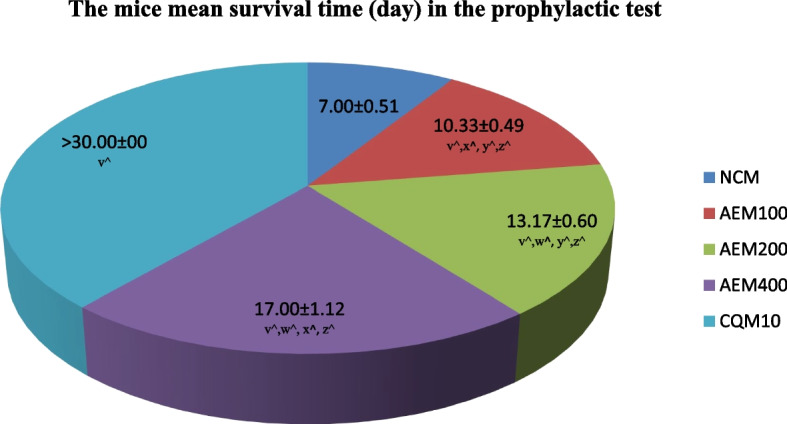
The measure of survival time (days) of mice provided various doses of the aqueous crude leaf extract of *C. sativum,* and then infected with *P. berghei* in the prophylactic test. Data presentation is in the form of mean ± standard error of the mean (Number of mice included in each of the group = 6). NCM, mice group which received negative control, distilled water, 10ml/kg/day; AEM100, mice group which received aqueous crude leaf extract, 100mg/kg/day; AEM200, mice group which received aqueous crude leaf extract, 200mg/kg/day; AEM400, mice group which received aqueous crude leaf extract, 400mg/kg/day; CQM10, mice group which received positive control, chloroquine base, 10mg/kg/day; v, comparing with NCM; w, comparing with AEM100; x, comparing with AEM200; y, comparing with AEM400; z, comparing with CQM10; ^*p* < 0.05. The mice were under close monitoring for a total of 30 days

Figure 9 describes the average survival in days of mice that were first provided increasing doses of aqueous crude leaf extract of *C. sativum* plant, and then infected with *P. berghei* in the prophylactic test. The longest average survival time for varying doses of the plant extract was found to be 17 days. Moreover, this effect was recorded for the group of mice that received 400mg/kg/day of the leaf extract. Throughout the monitoring period, 30 days, however, each of the mice which received CQ survived. The survival days for the mice group which received the vehicle were a week or less.

#### Influence on temperature and weight measures

Table 3 illustrates the mean change in percentages for weight and temperature of mice that were given differing doses of the aqueous leaf extract of *C. sativum* and infected with *P. berghei* in the chemopreventive antimalarial screening model. The plant extract inhibited the loss of weight occurring due to parasitemia development and averted the decline in temperature of the mice. In the negative control group, the percentage decline in temperature and weight were huge and found to be 7.03% and 5.56%, respectively. On the other hand, better improvements in both the parameters were seen with the upper dose of the extract and CQ. The percentage change in weight and temperature for the former was 10.28% and 3.25% in the respective order while it was 14.14% (increase) and 0.94% (decline) for the latter.

**Table 3:** Body weight and rectal temperature changes of mice provided various doses of the aqueous crude leaf extract of *C. sativum,* and then infected with *P. berghei* in the prophylactic test

Group	Body Weight (g)			Rectal Temperature (°C)		
	D_0_	D_4_	Change (%)	D_0_	D_4_	Change (%)
NC	25.82 ± 0.67	21.53 ± 0.40	-16.56 ± 0.68	36.50 ± 0.11	32.49 ± 0.28	-11.00 ± 0.62
AE100	26.07 ± 0.58	24.14 ± 0.62	-7.44 ± 0.52a^π^i^π^o^π^u^π^	36.18 ± 0.21	34.06 ± 0.18	-5.85 ± 0.92a^π^i^π^o^π^u^π^
AE200	25.96 ± 0.45	24.80 ± 0.43	-4.47 ± 0.37a^π^e^π^o^π^u^π^	36.42 ± 0.19	35.22 ± 0.23	-3.30 ± 0.23a^π^e^π^o^π^u^π^
AE400	26.02 ± 0.58	25.67 ± 0.62	-1.37 ± 0.45a^π^e^π^i^π^u^π^	36.60 ± 0.15	36.26 ± 0.14	-0.32 ± 0.13a^π^e^π^i^π^u^π^
CQ10	26.00 ± 0.75	26.42 ± 0.68	1.69 ± 1.20a^π^	36.52 ± 0.07	37.06 ± 0.13	1.48 ± 0.45a^π^

Measurements are expressed as mean ± standard error of the mean. (the number of mice assigned in a group = 6). *D*_*0*_ pre-treatment measured value on day 0, *D*_*4*_ post-treatment measured value on day 4, *NC* Negative control, distilled water, 10ml/kg/day, *AE* Aqueous crude leaf extract, *CQ* Chloroquine. a, comparing with NC; e, comparing with 100mg/kg; i, comparing with 200mg/kg; o, comparing with 400mg/kg; u, comparing with CQ10; ^π^*p* < 0.05. The numbers after the acronyms in the column titled group (400, 200, 100, 10) indicate the doses given to those groups in mg/kg/day

#### Influence on packed cell volume measure

**Fig. 10:**
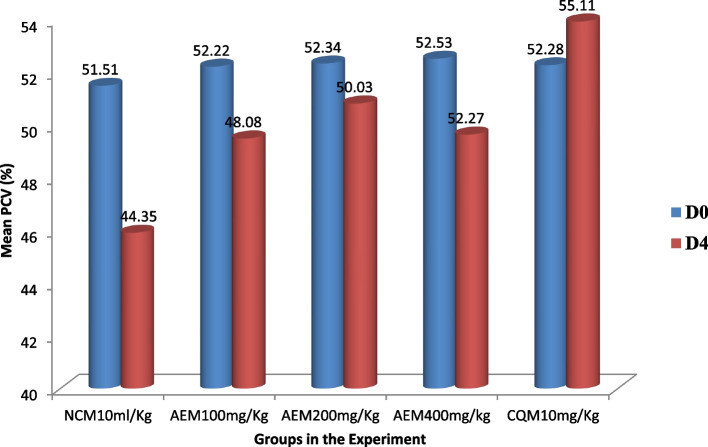
The measure of change of packed cell volume of mice provided various doses of the aqueous crude leaf extract of *C. sativum* and then infected with *P. berghei* in the prophylactic test. Data presentation is in the form of mean ± standard error of the mean (Number of mice included in each of the group = 6). D0, measure on day 0, pre-treatment; D4, measure on day 4, post-treatment. NCM10ml/kg, mice group which received negative control, distilled water, 10 ml/kg/day; AEM100mg/kg, mice group which received aqueous crude leaf extract, 100mg/kg/day; AEM200mg/kg, mice group which received aqueous crude leaf extract, 200mg/kg/day; AEM400mg/kg, mice group which received aqueous crude leaf extract, 400mg/kg/day; CQM10mg/kg, mice group which received positive control, chloroquine base, 10mg/kg/day. PCV, packed cell volume. a, comparing with NCM10ml/kg; e, comparing with AEM100mg/kg; i, comparing with AEM200mg/kg; o, comparing with AEM400mg/kg; u, comparing with CQM10mg/kg; ^π^*p* < 0.05. Indicated in the rectangles is the mean percentage change of PCV between post-treatment and pre-treatment measures

The aqueous crude leaf extract of the *C. sativum* plant prevented the malaria parasite- triggered decline of PCV relative to the vehicle in the chemophylactic antimalarial model (Fig. 10). The maximum mean percentage decline of PCV (10.80%) was measured in the mice group that received the distilled water. On the contrary, the lowest percentage reduction of PCV (2.86%) was recorded in those mice which received the extract at a dosage of 400mg/kg/day. Yet, this improvement in the hematologic profile was inferior to that of CQ where there was no decline in PCV from the base line. Significant difference (*p* < 0.05) in hematologic improvement was apparent in the comparison of the different doses of the extract and the standard drug with the solvent deployed as control.

#### Evaluation of secondary phytoconstituents

The investigation directed at determining the *C. sativum* plant leaf’s secondary phytochemicals showed that it contained alkaloids, flavonoids, glycosides, saponins and terpenoids. Alkaloids were found to be the most abundant constituents (14.32%). On the contrary, steroids and tannins were not observed (Table 4).

**Table 4:** Evaluation of the secondary phytochemical composition of the *C. sativum* plant aqueous crude leaf extract

Phytochemical composition	Outcome of the screening	Percentage of the constituent
Alkaloids	Po	14.32
Flavonoids	Po	7.04
Glycosides	po	4.23
Saponins	po	4.14
Steroids	pno	pno
Tannins	pno	pno
Terpenoids	po	2.56

*po* phytochemical observed, *pno* phytochemical not observed

## Discussion

Malaria is extremely endangering the socio-economic and health well-being of the international communities, especially pregnant women and children under 5 years dwelling in the third world [1, 10, 76]. The disease is taking a life of over half a million people each subsequent year. This has been aggravated by the inaccessibility and resistance of the parasite to affordable effective antimalarial medications. Most of the queue of antiplasmodial drugs used to combat the disease had lost their efficacy. For example, CQ was previously effective against all types of malaria parasites, but lost its efficacy against the severe form of malaria parasite *P. falciparum,* and most cases of *P. ovale*. Further, there are alarming reports including a recent report from Ethiopia regarding the development of resistance traits by malaria parasites to Artemisinins, the most effective drugs we currently have for the management of severe malaria caused by P*. falciparum *[3, 4, 27]. Hence, it is reasonable to conduct studies directed toward novel antimalarial drug discovery. Antimalarial drugs including Artemisinins were discovered through a thorough screening of folk medicinal plants. The efficacy study in animal models is the common trend towards the commencement of such investigations on traditional medicinal plants [24, 26, 77].

The common malaria screening models in animals encompass the four-day suppressive test, the curative test and the prophylactic test [65, 78]. The four-day suppressive test assesses the test substances’ antimalarial activity on early infection [64, 68], whereas the curative test is used to evaluate the antiplasmodial effect of the plant extract on established infection [30, 78]. The prophylactic model investigates the extract’s preventive capability against malarial disease development, unlike the former ones which assess the extract’s effect after disease develops and shows its manifestations [66, 72]. An ideal screening test for the evaluation of plant extract in early investigation shall involve multiple models measuring multiple parameters to be reliable. As a consequence, all the three antimalarial screening models with multiple parameters (weight, parasitemia, temperature, survival time, PCV) were employed in the present study.

The method for extraction of the plant, and *in vivo* Pharmacologic investigation on *C. sativum* to affirm its antiplasmodial effectiveness is logical. The reason for the *in vivo* test comes from the previous ethnobotanical survey that supports plant’s folk medicinal value [35]. Moreover, there was evidence that further augmented this traditional endowment, the in vitro chemosuppressive antiplasmodial activity of the herb [54, 55]. Plants with folkloric utilization in the management of the ailment, and *in vitro* positive inhibitory results, are candidates for tests in animal studies for further scientific approval [59, 60, 79]. The *in vitro* scientific test itself is not final in affirming the pharmacologic activity of a given herbal extract as it doesn’t consider any possible factors in living body such as the immune system, renal and hepatic enzymatic dispositions, and manipulations including chemical inactivation by endogenous compounds [58, 80]. In other words, *in vitro* suppressive activity is not a guarantee for the *in vivo* Pharmacologic effectiveness. The plant was extracted using distilled water to replicate the method employed by the traditional practitioners [35].

Application of the murine malaria model involving *P. berghei* infection in our study is in line with the standard protocol. Rodents such as mice are conducive animals for *in vivo* tests as they are mammals like humans to reproduce results and infer to humans. Furthermore, they can produce sufficient samples for investigation in a short period of time [81]. More importantly, previous *in vivo* Pharmacologic investigations leading to valuable medications employed mice in their practice. *P. berghei* was employed in our research work instead of human malaria parasites, the main reason being human malaria parasites’ inability to infect mice and lack of genetically modified humanized mice in our setup. Even though the use of realistic human parasites adds value, it is not necessarily indicated. This could be learned from most of the past investigations that utilized rodent malaria parasite *P. berghei* and led to the discovery of most of the antimalarial drugs, currently active and inactive, including artemisinins and quinine [26, 34].

Furthermore, an investigation on the CQ sensitive *P. berghei* is sensible because our aim herein the present study is to check for the Pharmacological effectiveness of the plant against malaria. Previous studies that discovered antimalarial drugs followed the same principle and found active compounds important for the resistance strains of the human parasites with better safety profile even if the gain from most of such weapons waned out over years [25, 34]. More importantly, this practice (model) is the already accepted standard [60, 82].

The findings from the four day suppressive, curative and chemoprophylactic tests indicated that the aqueous crude leaf extract of *C. sativum* is endowed with *in vivo* antiplasmodial activity in mice infected with *P. berghei*. Measure of parasitemia is said to be the most reliable indicator parameter for antimalarial efficacy of the plant extract in all the test models [64, 66, 83]. In this regard, the ceiling plasmodial suppression effect (82.74%) was elicited by the 400mg/kg/day of the extract in the four day suppressive test surpassing that of the curative test at the same dose by about 4%. This parasitemia suppressive effect was similar to the investigations conducted on *Euclea divinorum *[66]*, Maytenus gracilipes *[83]*, Annona muricata *[69]*,Fadogia ciekowskii, Myrianthus libericus *[84]*,Lophira lanceolate,Vernonia conferta, Protea madiensis *[85]*,* and *Spondias pinnata *[74] in which the suppression was better in the four-day test. On the contrary, the curative test yielded better parasitemia suppression unlike our study in researches done on *Eucalyptus camadulensis *[86]*,* and *Balanites rotundifolia *[78]. In the prophylactic evaluation of the plant extract, however, the highest parasite suppressive effect recorded was 50.09% even at 400mg/kg/day dosing. This finding is in line with work on *Spondias pinnata *[74] where the prophylactic chemosuppression was the least while it contradicts the study done on *Piliostigma reticulatum *[87] where the plant showed the highest effect in prophylactic test.

Moreover, the plant extract demonstrated a statistically significant inhibition of parasitemia (*p* < 0.05) relative to the vehicle in all the three models where higher doses resulted in better plasmodial inhibition activity. The maximum parasite suppression capability of our study was also compared with previous works done by different authors on other plants. Accordingly, the parasitemia suppression of the plant under investigation herein is superior to the study done on *Terminalia arjuna* (67.95%) [30], *Spondias pinnata* (66.82%) [74], *Lobelia giberroa* (73.05%) [88], *Alstonia boonei* (68.35%) [89], *Schinus molle* (66.91%) [60],,*Lagenaria siceraria (*77.37% %) [58]*, Maytenus gracilipes* (74.15%) [83], *Saccharum officinarum* (70.20%) [90], *Phyllanthus emblica, Terminalia chebula, Terminalia bellerica* (53.40% to 69.46%) [91], *Canthium multiflorum* (81.90%) [92], *Myrianthus libericus* (77.11%) [84]*, Spondias pinnata* (66.82%) [74], *Vernonia Amygdalina* (42.26%) [93] and *Balanites rotundifolia* (69%) [78]*.* Nevertheless, the findings of our work on the leaf extract of *C. sativum* pertaining to parasitemia suppression was nearly equal or inferior to the work done on Artemisia abyssinica (82.4%), [94] *Eucalyptus camadulensis (95.30%) *[86], *Senna alata* (84.36%), *Capsicum frutescens)(93.28%, Moringa oleifera (95.6%) *[95]*, Avicennia marina(93%) *[96] and *Ficus thoningii (91%) *[97]. None of the studies mentioned in this text including this experimental investigation cleared the parasite unlike the standard antimalarial drug, CQ (100% suppression). The reason for the incomplete eradication of the parasite by the extracts could be short half-life of the active components that would have required frequent dosing. Otherwise, the active ingredient/s in the plants might require further structural modifications to be potent drugs. Metabolic elimination, immune system, or the yet undiscovered mechanism would be involved [60].

According to literature reports, antiplasmodial agent eliciting a level of parasitemia less than/equal to 90% for the negative control, infers that the test substance is active antimalarial candidate in standard antiplasmodial screening procedures. As the values are less than the mentioned cut points, the aqueous leaf extract of *C. sativum* is considered an active antiplasmodial chemosuppressive agent. In addition, a potential antimalarial candidate should exhibit percentage parasitemia suppression of greater than/equal to 30%. Again, the aqueous extract of *C. sativum* satisfies this criterion (82.74% suppression), so the plant is a malaria-destroying arsenal. Evidence from literature sources also shows that the percent parasitemia inhibition of ≥ 50% at different dose ranges of the plant extract can be rated for their degree of activity: 500–250 mg/kg/day (moderate), 250–100 mg/kg/day (good), ≤ 100 mg/kg/day (very good). As a result, the leaf extract of *C. sativum* is endowed with a very good (51.72% at 100 mg/kg/day) malaria-relieving effect [64].

The antiplasmodial effect exerted by the aqueous extract of *C. sativum* could be due to the abundant phytochemicals localized in the plant. The findings from the secondary phytochemical profiling indicated that the plant contained alkaloids, flavonoids, glycosides, saponins and terpenoids. The terpenoids and flavonoids contained in the plant showed anti-oxidant, free radical scavenging and anti-inflammatory activities according to the studies previously conducted on the plant [98]. These host protective actions could be responsible for the antiplamodial property exerted by the extract. Moreover, the plant is rich in the alkaloid constituents (14.32%). The alkaloids have recorded historical evidence as conventional antimalarial drugs, quinine from cinchona bark being a prototype [99, 100]. These profusely concentrated alkaloidal compounds in the plant leaf could be the major reason for the antiplasmodial efficacy of the extract. Still, the immune system might be boosted by these components to tackle the development of the malaria-causing parasite in the body. Also, inhibition of the pathways in the malaria reproductive cycle in the vertebrates could be the other suggested mechanism for ameliorating malaria parasite- induced harm [6, 58, 82].

In addition to the measure of parasitemia discussed above, PCV, body weight, survival time and temperature were recorded and assessed in each of the models. Inhibition of hypothermia, weight loss hampering, improved survival and protection against hemolysis were elicited by the extract as evidenced from the findings. This is in line with the general consensus that a candidate antimalarial plant extract is expected to halt weight loss, prevent temperature fall, inhibit RBCs hemolysis and extend the survival life of mice under the condition of malaria infection [82, 83, 96, 101]. Comparted to the negative control, extract’s anemia inhibiting effect, degree of resistance to weight loss, and hypothermia were statistically significant (*p* < 0.05). The inhibition of weight loss and PCV decline exhibited by the extract was dose-dependent where higher doses better counteracted the decline of these parameters. Nonetheless, even the highest dose administered didn’t absolutely produce zero or positive percentage difference in both the parameters. Previous works suggested that recrudesce and short duration of action for the active components resulting from the increased metabolism and elimination could be responsible for this pitfall [64, 66]. Comparing the weight and PCV protection capability of the extract among the three models, it was found that the plant’s percentage inhibition was higher in the four-day suppressive test in line with the parasitemia suppression. This effect is similar to the researches on *Vernonia amygdalina* [93] *and Maytenus gracipiles *[83]. On the other hand, the findings on *Balanites rotundifolia *[78] showed the plant’s pronounced effect on weight and PCV in curative test. In the present study, the lowest percentage inhibition was scored in the chemoprophylactic test. This finding is similar to the research work done on *Saccharum officinarum *[90].

In the plant’s screening for the antimalarial potential, mice survival time is also among the widely monitored parameters [78, 82]. It is agreed up on that the candidate extract should extend the survival time of mice in comparison to the negative control substance. In accordance with this, in all the three models, the extract was found to prolong the survival time of the test mice better than the negative control mice, which is also statistically significant (*p* < 0.05). Mice that were administered the extract to assess the effect of the substance in early infection survived more days than those in the curative test, while the short survival days were measured among those in prophylaxis procedure. The effect on the MST is similar to previous research where the survival time is longer in the four day suppressive test [83]. However, in none of the models the mean survival of the mice was reported to be 30 during the monthly monitoring period. This could be attributed to the suboptimal clearance of the parasites from blood due to the above-mentioned reasons in this document. Contrary to this, the MST of mice that received the standard antimalarial drug was greater than or equal to 30 days because of the complete eradication of the blood parasite, plasmodium. Eventually, this measure and the aforementioned parameters indicate that the antiplasmodial effect of the aqueous crude leaf extract of *C. sativum* was more pronounced on the early and established infection than the prophylaxis test.

The investigation on plant’s safety establishment in acute toxicity study, up on observation for gross behavioral changes and physical appearances, revealed that no obvious symptoms of acute poisoning were elicited by the plant extract. In addition, during the first-day single animal test, and throughout the acute toxicity study follow-up period (two weeks), the death of any of the experimental mice was not seen at the administered dose level of 2000 mg/kg. Hence, the median lethal dose (LD_50_) of the leaf extract from *C. sativum* could exceed 2000 mg/kg according to the OECD guideline No 425 [102]. Thus, the safe use of *C. sativum* by the Ethiopian folk for malaria management is reasonable as supported by these test findings.

### Limitation pertaining to the current experimental work

The present study thrived to dig out the antimalarial effect of the crude extract of the plant employing different models and multiple parameters. However, the effect of the plant with different solvents was not explored. Further, the mode of action of the plant extract was not investigated. Identification of specific bioactive compounds responsible for eliciting antimalarial activity was not achieved. Hence, future studies focus on the utilization of state of-the-art technologies for the identification, isolation, characterization and structural elucidation of such invaluable compounds localized in the plant.

## Conclusion

The results of our experimental study revealed that the aqueous crude leaf extract of *C. sativum* exhibits significant antimalarial efficacy in multiple in vivo models involving mice infected with *P. berghei*. Notably, the extract demonstrated a substantial parasite inhibition of up to 82.74%. Additionally, it effectively mitigated hypothermia, attenuated weight loss, improved survival outcomes, and conferred protection against hemolysis. Given these promising therapeutic attributes, in depth investigation on the plant is recommended.

## Data Availability

The datasets used and/or analysed during the current study are available from the corresponding author on reasonable request.

## Abbreviations

ANOVA: One Way Analysis of Variance
CQ: Chloroquine
EPHI: Ethiopian Public Health Institute
MST: Mean Survival Time
OECD: Organization for Economic Cooperation and Development
PCV: Packed Cell Volume
PP: Percentage Parasitemia
PPS: Percentage Parasitemia Suppression
SEM: Standard Error of the Mean
SPSS: Statistical Package for Social Sciences
WHO: World Health Organization
