# Correction: Antimalarial efficacy test of the aqueous crude leaf extract of *Coriandrum sativum* Linn.: an *in vivo* multiple model experimental study in mice infected with *Plasmodium berghei*

**DOI:** 10.1186/s12906-024-04598-9

**Published:** 2024-08-01

**Authors:** Getu Habte, Sisay Habte, Oda Jilo, Wondwosen Alemu, Kedir Eyasu, Welela Meka, Getabalew Shifera, Wubishet Gezimu, Milkias Dugasa, Sanbato Tamiru, Meta Mamo, Abiyot Kelecha

**Affiliations:** 1 Department of Pharmacology and Clinical Pharmacy, School of Pharmacy, College of Health Sciences, Addis Ababa University, P.O. Box 1176, Addis Ababa, Ethiopia; 2 Department of Pharmacy, College of Health Sciences, Mattu University, P.O. Box 318, Mettu, Ethiopia; 3 Department of Biology, College of Natural and Computational Sciences, Ambo University, P.O. Box 19, Ambo, Ethiopia; 4 Department of Computer Science, College of Engineering and Technology, Mattu University, P.O. Box 318, Mettu, Ethiopia; 5 Department of Chemistry, College of Natural and Computational Sciences, Mattu University, P.O.Box 318, Mettu, Ethiopia; 6 Department of Nursing, College of Health Sciences, Mattu University, P.O. Box 318, Mettu, Ethiopia

**Correction: BMC Complement Med Ther 24, 267 (2024)**

10.1186/s12906-024-04577-0

Following publication of the original article [1], the authors reported an error in the author name of Abiyot Kelecha.

The incorrect author name is Abiyo Kelecha.

The correct author name is Abiyot Kelecha.

The author group has been updated above and the original article [1] has been corrected.
